# A New Sesquilignan Glucoside from *Uraria sinensis*

**DOI:** 10.3390/molecules19011178

**Published:** 2014-01-17

**Authors:** Yingda Yang, Zhengxi Hu, Zengwei Luo, Yongbo Xue, Guangmin Yao, Yanyan Wang, Yonghui Zhang

**Affiliations:** 1 Hubei Key Laboratory of Natural Medicinal Chemistry and Resource Evaluation, School of Pharmacy, Tongji Medical College, Huazhong University of Science and Technology, Wuhan 430030, China; 2 First College of Clinical Medical Science of China, Three Gorges University & Yichang Central People’s Hospital, Yichang 443003, China

**Keywords:** *Uraria sinensis*, sesquilignan glucoside, lignans, flavonoids

## Abstract

A new sesquilignan glucoside, urariasinoside A (**1**), together with eight known compounds, including two lignans, a sesquilignan, a dilignan, and four flavonoid derivatives were isolated from the aerial parts of *Uraria sinensis*. Their structures were determined on the basis of extensive spectroscopic analyses and comparison with literature data. Compound **1** was evaluated for *in vitro* cytotoxicity activity against HL-60, SMMC-7721, A549, MCF-7, SW480, and BEAS-2B cell lines.

## 1. Introduction

**Figure 1:**
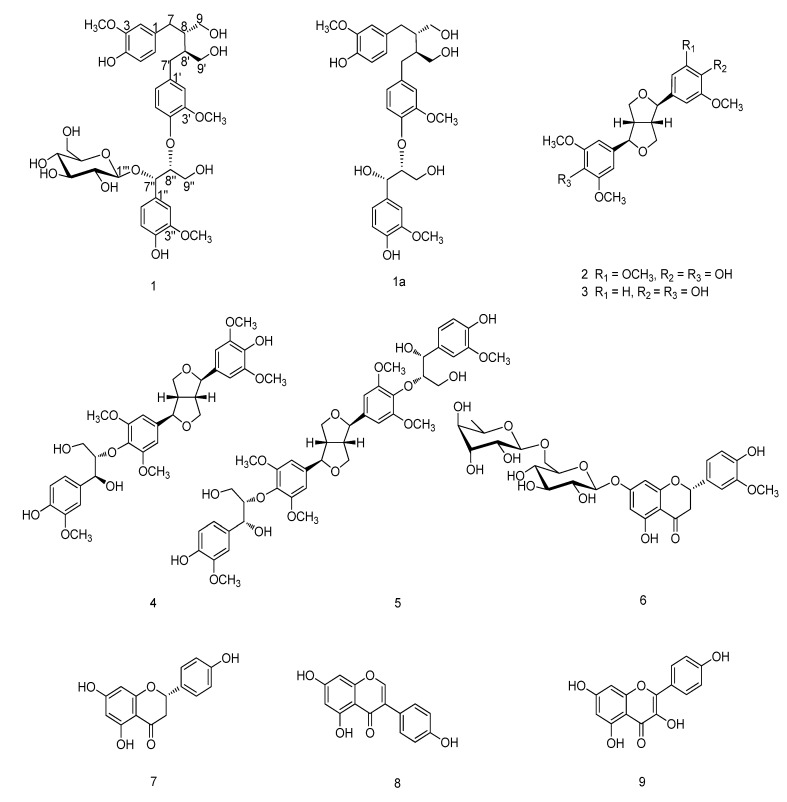
Structures of compounds **1**–**9**.

The genus *Uraria* (family Leguminosae), consists of 35 species, distributed throughout the tropical regions of Africa, Asia, and Australia [1]. Traditionally, some species of this genus are used as folk medicines for the treatment of gonorrhea, cough, chills and fever [2], or as therapies for swelling, coldness, ulcers and stomachalgia [3]. Pharmacological investigations have revealed that the extract of *U*. *lagopoides* has anti-inflammatory and analgesic activities [4], *U*. *critina* exerts nitric oxide-scavenging and antioxidant effects [5], and *U*. *picta* has antimicrobial activity [6]. Previous phytochemical studies on this genus reported the presence of isoflavanones, triterpenes, steroids, glycosides, and aromatic components [6,7,8]. *Uraria sinensis* Desv. (Hemsl.), a suffruticose sparingly branched perennial herb, is exclusively distributed in the Hubei, Sichuan, Guizhou, Yunnan, Shanxi, Gansu provinces of China [1]. To date, this species has not been investigated. As part of our ongoing search for bioactive natural compounds from the genus *Uraria*, we report the isolation and structure elucidation of a new sesquilignan glucoside, urariasinoside A (**1**), together with eight known compounds, including two lignans, a sesquilignan, a dilignan, and four flavonoid derivatives from the aerial parts of *U. sinensis* (Figure 1), as well as the inhibitory activities against five human cancer cell lines (HL-60, SMMC-7721, A549, MCF-7, and SW480) and a normal cell line (BEAS-2B) of compound **1**.

## 2. Results and Discussion

The EtOH extract of the aerial parts of *U. sinensis* was suspended in water and successively partitioned with petroleum ether, CH_2_Cl_2_, and *n*-butanol. The *n*-butanol-soluble extract was subjected to column chromatography to afford a new sesquilignan glucoside, urarisinoside A (**1**), along with a known compound, 5,4'-dihydroxy-3'-methoxyflavanone-7-(6''*O*-*β*-l-rhamnopyranosyl)-*β*-D-glucopyranoside (**6**) [9]. The CH_2_Cl_2_-soluble extract was subjected to repeated column chromatography to yield seven known compounds: (–)-syringaresinol (**2**) [10], (–)-medioresinol (**3**) [11], (–)-(7*R*,7'*R*,7''*S*,8*S*,8'*S*,8''*S*)-4',4''-dihydroxy-3,3',3'',5,5'-pentamethoxy-7,9':7',9-diepoxy-4,8''-oxy-8,8'-sesquineolignan-7'',9''-diol (**4**) [12], (+)-(7*R*,7'*R*,7''*R*,7'''*R*,8*S*,8'*S*,8''*S*,8'''*S*)-4'',4'''-dihydroxy-3,3',3'',3''',5,5'-hexamethoxy-7,9':7',9-diepoxy-4,8'':4',8'''-bisoxy-8,8'-dineolignan-7'',7''',9'',9''-tetraol (**5**) [12], naringenin (**7**) [13], quercetin (**8**) [14], and kaempferol (**9**) [14]. The known compounds were identified on the basis of NMR spectroscopic analyses and comparison with the data reported in the literature.

Compound **1** was obtained as pale yellow oil with the molecular formula C_36_H_48_O_15_, as evidenced by a pseudo-molecular ion peak [M+Na]^+^ at *m/z* 743.2863 in the HRESIMS spectrum. The UV spectrum suggested the existence of conjugated groups on the basis of maximum absorption bands at 228 nm and 280 nm. The IR spectrum exhibited the presence of hydroxyl groups (3364 cm^−1^) and benzene rings (1603 cm^−1^ and 1513 cm^−1^). Its ^1^H-NMR spectrum (Table 1) showed three ABX spin systems assignable to three sets of 1,3,4-trisubstituted aromatic rings at *δ*_H_ 6.59 (1H, d, *J* = 1.1 Hz), 6.63 (1H, d, *J* = 8.0 Hz), and 6.50 (1H, dd, *J* = 8.0, 1.1 Hz); 6.61 (1H, d, *J* = 1.0 Hz), 6.70 (1H, d, *J* = 8.1 Hz), and 6.53 (1H, dd, *J* = 8.1, 1.0 Hz); 7.12 (1H, d, *J* = 1.1 Hz), 6.74 (1H, d, *J* = 8.1 Hz), and 6.86 (1H, dd, *J* = 8.1, 1.1 Hz). Its ^13^C-NMR and DEPT spectral data (Table 1) revealed the presence of 27 carbons except for the carbon signals due to a glucopyranose moiety (*δ*c 101.1, 75.3, 77.8, 71.9, 77.8, and 62.8) and three methoxy groups (*δ*_C_ 56.5, 56.4, and 56.4). Subsequently, 27 carbons were assigned to a sesquilignan moiety, including three groups of aromatic carbons: nine quaternary carbons (*δ*_C_ 151.4, 149.1, 148.9, 147.6 147.5, 145.6, 136.9, 133.9, and 130.5), and nine unsubstituted aromatic carbons (*δ*_C_ 122.8, 122.8, 122.0, 118.4, 116.0, 115.9, 114.0, 113.6, and 112.8). The remaining 9 carbons were ascribed to five methylene carbons (*δ*_C_ 62.2, 62.1, 61.9, 36.1, and 36.0), and four methine carbons (*δ*_C_ 86.1, 77.9, 44.4, and 44.0). These before mentioned ^1^H- and ^13^C-NMR data implied that compound **1** should be a sesquilignan glucoside.

**Figure 2:**
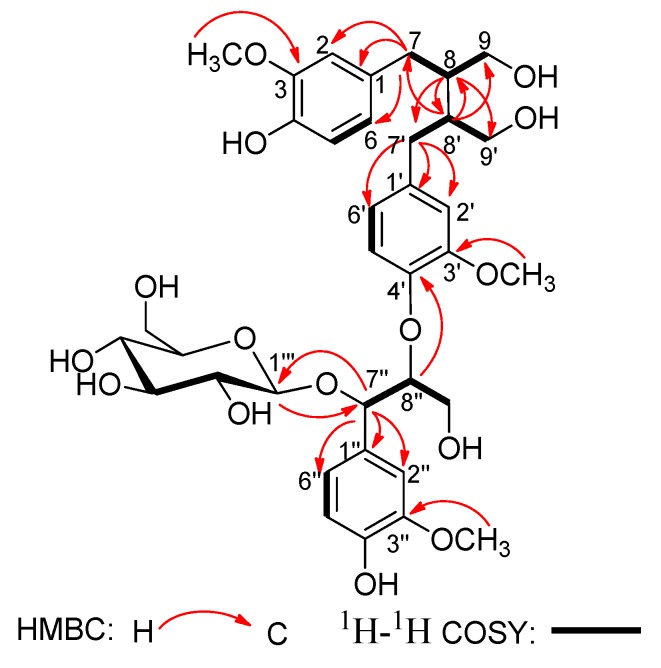
Key HMBC and ^1^H-^1^H COSY correlations of compound **1**.

In the ^1^H-^1^H COSY spectrum (Figure 2), the consecutive cross-peaks starting from the methylene protons at *δ*_H_ 2.52 (1H, m, H-7a) and 2.62 (1H, m, H-7b) to methine proton at *δ*_H_ 1.87 (1H, m, H-8), and from H-8 to the methylene protons at *δ*_H_ 3.56 (1H, dd, *J* = 11.2, 4.3Hz, H-9a) and 3.82 (1H, dd, *J* = 11.2, 5.1 Hz, H-9b) suggested the linkage of the C_3_ fragment of ^7^CH_2_-^8^CH-^9^CH_2_OH. The ^1^H–^1^H COSY correlation of CH_2_-7' with CH-8' and the correlation of CH-8' with CH_2_-9' revealed the connectivity of the C_3_ unit ^7'^CH_2_-^8'^CH-^9'^CH_2_OH. Additionally, the ^1^H-^1^H COSY correlation of the oxygenated methine proton at *δ*_H_ 5.17 (1H, d, *J* = 4.0 Hz, H-7'') with the oxygenated methine proton at *δ*_H_ 4.30 (1H, m, H-8''), and the correlation of H-8'' with the oxygenated methylene protons at *δ*_H_ 3.51 (1H, dd, *J* = 11.1, 5.1 Hz, H-9''a) and 3.84 (1H, dd, *J* = 11.1, 4.2 Hz, H-9''b) disclosed the fragment of the third the C_3_ unit ^7''^CH(O)-^8''^CH(O)-^9''^CH_2_OH. These three C_3_ units were connected to the above three sets of 1,3,4-trisubstituted phenyl groups respectively, according to the HMBC correlations (Figure 2) of H-7 with C-1, C-2, and C-6; of H-7' with C-1', C-2', and C-6'; and of H-7'' with C-1'', C-2'', and C-6''.

**Table 1:** ^1^H-NMR (400 MHz) and ^13^C-NMR (100 MHz) data of compound **1** (in CD_3_OD, *δ* in ppm, *J* in Hz).

NO.	*δ*_H_	*δ*_C_	NO.	*δ*_H_	*δ*_C_
1	-	133.9	9'b	3.56 dd (11.2, 4.3)	-
2	6.59 d (1.1)	113.6	1''	-	130.5
3	-	149.1	2''	7.12 d (1.1)	112.8
4	-	145.6	3''	-	148.9
5	6.63 d (8.0)	116.0	4''	-	147.6
6	6.50 dd (8.0, 1.1)	122.8	5''	6.74 d (8.1)	115.9
7a	2.52 m	36.0	6''	6.86 dd (8.1, 1.1)	122.0
7b	2.62 m	-	7''	5.17 d (4.0)	77.9
8	1.87 m	44.0	8''	4.30 m	86.1
9a	3.56 dd (11.2, 5.1)	62.1	9''a	3.51 dd (11.1, 5.1)	61.9
9b	3.82 dd (11.2, 4.3)	-	9''b	3.84 dd (11.1, 4.2)	-
1'	-	136.9	Glc-1'''	4.15 d (7.4)	101.1
2'	6.61 d (1.0)	114.0	2'''	3.32 dd (8.9, 7.4)	75.3
3'	-	151.4	3'''	3.27 t (8.9)	77.8
4'	-	147.5	4'''	3.28 m	71.9
5'	6.70 d (8.1)	118.4	5'''	3.11 m	77.8
6'	6.53 dd (8.1, 1.0)	122.8	6'''a	3.66 dd (11.8, 4.5)	62.8
7'a	2.52 m	36.1	6'''b	3.83 dd (11.8, 2.3)	-
7'b	2.62 m	-	3-OCH_3_	3.69 s	56.4
8'	1.86 m	44.4	3'-OCH_3_	3.69 s	56.4
9'a	3.52 dd (11.2, 5.1)	62.2	3''-OCH_3_	3.76 s	56.5

**Figure 3:**
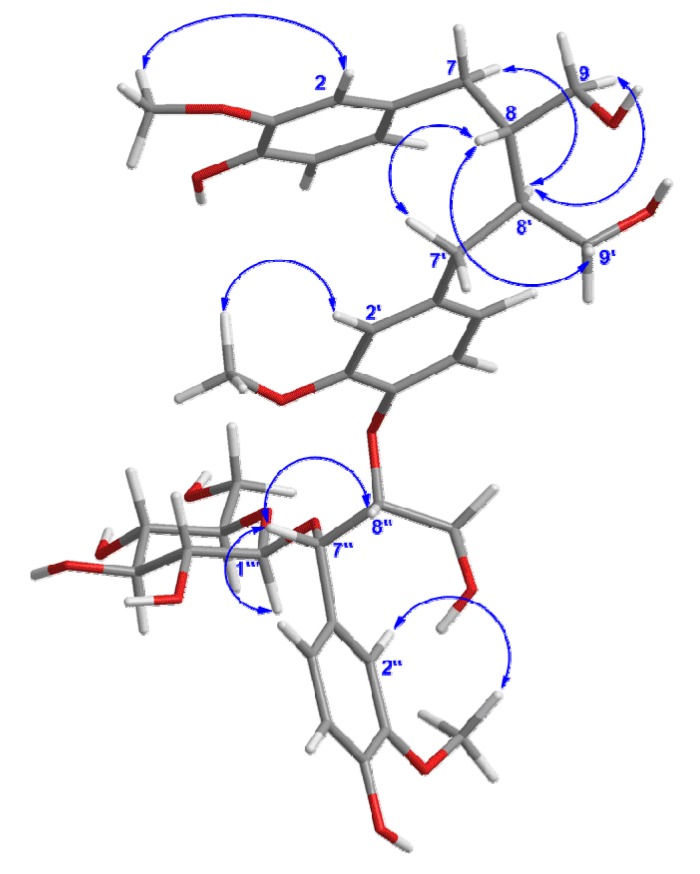
Key NOESY correlations of compound **1**.

Moreover, the HMBC correlation (Figure 2) between H-8'' and C-4' verified ether linkage between C-8'' and C-4'. Meanwhile, the C-8/C-8' linkage was confirmed by HMBC correlations of H-8 with C-7', C-8' and C-9', and of H-8' with C-7, C-8 and C-9 in the HMBC spectrum. The signals due to methoxy protons were assigned as 3-OCH_3_, 3'-OCH_3_, and 3''-OCH_3_, on the basis of the cross-peaks from H-3-OCH_3_ to C-3, H-3'-OCH_3_ to C-3', and H-3''-OCH_3_ to C-3'' according to the HMBC spectrum. The position of 3-OCH_3_, 3'-OCH_3_, and 3''-OCH_3_ was also corroborated by the correlations between H-3-OCH_3_ and H-2, H-3'-OCH_3_ and H-2', and H-3''-OCH_3_ and H-2'' in the NOESY spectrum (Figure 3). Thus, all the information mentioned above suggested that compound **1** was a secoisolariciresinol-sesquilignan derivative related to sesquimarocanol B [15,17,18].

The remaining proton signals from *δ*_H_ 3.83 to 3.32 in the ^1^H-NMR spectrum disclosed a set of *β*-glucopyranose moiety with the anomeric proton resonated at *δ*_H_ 4.15 (1H, d, *J* = 7.4 Hz, H-1'''), corresponding to six carbon signals at *δ*c 101.1, 75.3, 77.8, 71.9, 77.8, 62.8 in ^13^C-NMR spectrum. The *β*-glucose was located at C-7'' as elucidated by the HMBC correlation between Glc-H-1''' and the oxygenated methine carbon C-7'', which was further confirmed by the NOESY correlation between H-1''' and H-7''. Herein, **1** was identified as a secoisolariciresinol-sesquilignan glucoside. Furthermore, the presence of *β*-D-glucose was determined by GC analysis of its trimethylsilyl thiazolidine derivative after acid hydrolysis [16].

The relative configuration of **1** was elucidated by the NOESY (Figure 3) experiment, together with comparison with data of sesquimarocanol B. Likewise, the NOESY correlations of H-8' with H-7 and H-9, of H-8-with H-7' and H-9', and the absence of the NOESY cross-peak between H-8 and H-8', pointed to the *trans* orientations for H-8/H-8' as that of sesquimarocanol B. Similarly, the relative stereochemistry of H-7''/H-8'' was supposed to *cis* configuration according to the correlation between H-7'' and H-8''.

Enzymatic hydrolysis of **1** by the cellulase liberated an aglycon **1a**, which had the molecular C_30_H_38_O_10_, as deduced from HRESIMS at *m/z* 581.2344 [M+Na]^+^. The NMR data of **1a** was very similar with that of sesquimarocanol B [15]. However, the coupling constants of H-7''/H-8'' for **1a** and sesquimarocanol B were obviously different. In sesquimarocanol B, a large coupling constant (*J* = 6.3 Hz) suggested the *threo* configuration of H-7''/H-8'' [17]. Whereas in **1a**, a small coupling constant (*J* = 4.0 Hz) of H-7''/H-8'', and the proton signal of 7'' at *δ*_H_ 4.88 further confirmed an *erythro* configuration between H-7''/H-8'' in **1**, as a stereoisomer of sesquimarocanol B [19,20,21]. In addition, the absolute configuration of C-8'' was determined to be *R*, as the CD spectrum showed a negative Cotton effect at 239 nm [22,23,24]. Correspondingly, the absolute configuration of 7'' was elucidated as *S*. Finally, the structure of **1** was determined to be (7''*S*,8''*R*)-(–)-4,4'',9,9',9''-pentahydroxy-3,3',3''-trimethoxy-4',8''-oxy-8,8'-sesquineolignan-7''-*O*-*β*-D-glucopyranoside, to which the trivial name urariasinoside A was assigned.

In the other respects, previous reports of cytotoxicity activity of secoisolariciresinol-sesquilignan related structures such as sesquimarocanol B hexaacetate [16], suggest compound **1** might have potential activity as an anticancer agent. Therefore, compound **1** was evaluated for its cytotoxic activities against five human cancer cell lines: HL-60 (human myeloid leukemia), SMMC-7721 (hepatocellular carcinoma), A549 (lung), MCF-7 (breast), and SW480 (colon) by the MTT method [25]. DDP (cisplatin) and taxol were used as positive controls. However, the bioassay results revealed that compound **1** has no *in vitro* cytotoxicity (IC_50_ > 40 μM) against any of the five tested cancer cell lines.

## 3. Experimental

### 3.1. General Procedures

Optical rotations were measured on a Perkin Elmer PE-341LC polarimeter. UV spectra were measured on a Perkin Elmer Lambda 35 spectrometer. CD spectra were recorded on a JASCO J-810 CD spectrometer. IR spectra were recorded on a Bruker Vertex 70 FT-IR microscope instrument (FT-IRmicroscope transmission). NMR spectra were obtained at 400 MHz for ^1^H and 100 for ^13^C, on Bruker AM-400 MHz spectrometers with solvent peaks being used as references. Two-dimensional HSQC, HMBC, COSY, and NOESY experiments were performed using the pulse sequences provided by Bruker. HRESIMS data were measured using an API QSTAR Pulsar spectrometer. Column chromatography was performed using polyamide (10–30 mesh, Taizhou Luqiao Sijia Biochemical Plastics Factory, Taizhou, China), silica gel (100–200, 200–300 mesh, H, Qingdao Marine Chemical Inc., China), ODS (50 μm, YMC, Kyoto, Japan) and Sephadex LH-20 (Pharmacia Biotech AB, Uppsala Sweden). HPLC separation was performed on an instrument consisting of an Ultimate 3000 controller, an Ultimate 3000 pump, and an Ultimate 3000 UV detector with an YMC (250 × 10 mm, 5 μm) preparative column. GC analysis was performed on an Agilent Technologies 7820A GC instrument, OV-17 column (30 m × 0.32 mm × 0.5 μm, Lanzhou Zhongke Antai Analysis Technology Co. Ltd., Lanzhou, China), hydrogen-flame ionization detector. Enzymatic hydrolysis was treated with cellulase (Shanghai Yuanye Biology & Technology Co. Ltd., Shanghai, China). TLC was carried out on precoated silica gel GF254 plates. Spots were visualized under UV light (254 or 356 nm) or by spraying with 5% H_2_SO_4_ in 95% EtOH followed by heating.

### 3.2. Plant Material

The aerial parts of *U. sinensis* were collected from Shiyan, Hubei Province, China in September 2010 and identified by Dr. Jianping Wang of School of Pharmacy, Tongji Medical College, Huazhong University of Science and Technology. The voucher specimen (1009) was deposited in the herbarium of Hubei Key Laboratory of Natural Medicinal Chemistry and Resource Evaluation, School of Pharmacy, Tongji Medical College, Huazhong University of Science and Technology.

### 3.3. Extraction and Isolation

The air-dried aerial parts of *U. sinensis* (45 kg) were extracted three times with 95% EtOH (50 L) at room temperature for 24 hThe dried EtOH extract (4.0 kg) was suspended in H_2_O and then partitioned successively with petroleum ether (10.0 L × 3), CH_2_Cl_2_ (10.0 L × 3) and *n*-BuOH (10.0 L × 3). The *n*-butanol portion (375 g) was fractionated by column chromatography over polyamide (6 Kg) using H_2_O–EtOH of increasing polarity (100:0 (50 L × 3), 10:90 (50 L × 3), 30:70 (50 L × 3), 0:100 (50 L × 3), *v/v*). The eluates were combined together on the basis of TLC analysis. Then, the fractions eluted with pure water (the extract 172 g) were further subjected to silica gel column chromatography (CHCl_3_–MeOH–H_2_O 15:1:0 to 3:1:0.1, *v*/*v*/*v*) to afford seven fractions (Fr.1–7). Fr.6 (11.7 g) was subjected to silica gel column chromatography (CHCl_3_–MeOH–H_2_O 5:1:0.1 to 3:1:0.1, *v*/*v*/*v*) once again to give four subfractions (Fr.6.1–6.4). Fr.6.3 was subjected to a Sephadex LH-20 column eluted with MeOH to give six subfractions (Fr.6.3.1–6.3.6). Fr.6.3.4 was purified by silica gel H column chromatography (CHCl_3_–MeOH–H_2_O 3:1:0.1, *v*/*v*/*v*) to obtain compound **6** (10.5 mg). Fr.6.3.3 was subjected to ODS column chromatography eluted with 50% MeOH in H_2_O, and then purified by semi-preparative HPLC (37% MeOH in H_2_O, flow rate 1.8 mL/min, wavelength 212 nm) to yield compound **1** (5.0 mg, retention time 37 min).

The CH_2_Cl_2_ extract (150 g) was applied to silica gel column chromatography eluted with petroleum ether–acetone (10:1 to 1:1, *v*/*v*) to afford ten fractions (Fr.A–J). Compound **10** (20.0 mg) was recrystallized from Fr.G using acetone. Fr.G (5.1 g) was subjected to silica gel column chromatography (CHCl_3_–MeOH 20:1 to 8:1, *v*/*v*) to afford seven subfractions (Fr.G_1_–G_7_). Fr.G_3_ and Fr.G_5_ were subjected to a Sephadex LH-20 column eluted with CH_2_Cl_2_–MeOH (1:1, *v*/*v*) to give compound **8** (16.4 mg) by recrystallization. Compound **9** (14.5 mg) was obtained by recrystallization in acetone from Fr.G_3_.

Fr.H (20.0 g) was subjected to silica gel column chromatography (CHCl_3_–MeOH 30:1: to 8:1, *v*/*v*) to afford eight subfractions (Fr.H_1_–H_8_). Fr.H_2_ was applied to MCI gel column eluted with MeOH/H_2_O (8:1, *v*/*v*), and then was subjected to a Sephadex LH-20 column eluted with MeOH to give seven subfractions (Fr.H_2.1–2.7_). Fr.H_2.2_ was further purified by semi-preparative HPLC (MeOH–MeCN–H_2_O 35:15:50, flow rate 2.5 mL/min, wavelength 254 nm) to yield compound **2** (17.5 mg, retention time 20 min) and compound **3** (8.0 mg, retention time 23 min).

Fr.I (6.0 g) was subjected to silica gel column chromatography (CHCl_3_–MeOH 15:1: to 7:1, *v*/*v*) to afford nine subfractions (Fr.I_1_–I_9_). Fr.I_1_ was subjected to a Sephadex LH-20 column eluted with CH_2_Cl_2_–MeOH (1:1, *v*/*v*) to give four subfractions (Fr.I_1.1–1.4_). Fr.I_1.2_ was subjected to ODS column chromatography eluted with 50% MeOH in H_2_O, and then purified by semi-preparative HPLC (MeOH–MeCN–H_2_O 20:18:62, flow rate 2.5 mL/min, wavelength 212 nm) to yield compound **4** (7.2 mg, retention time 58 min).

Fr.J (25.0 g) was subjected to silica gel column chromatography (CHCl_3_–MeOH 12:1 to 8:1, *v*/*v*) to afford four subfractions (Fr.J_1_–J_8_). Fr.J_2_ was subjected to a Sephadex LH-20 column eluted with CH_2_Cl_2_–MeOH (1:1, *v*/*v*) to remove chlorophyll, and then further subjected to ODS column chromatography eluted with 50% MeOH in H_2_O to give two subfractions (Fr.J_2.1–2.2_). Fr.J_2.1_ was subjected to silica gel column chromatography (CHCl_3_–Me_2_CO–MeOH 30:15:0.1, *v*/*v*/*v*) and then purified by semi-preparative HPLC (MeOH–MeCN–H_2_O 29:21:50, flow rate 2 mL/min, wavelength 254 nm) to yield compound **5** (2.3 mg, retention time 30 min).

*Urariasinoside A [(7''S,8''R)-(–)-4,4'',9,9',9''-pentahydroxy-3,3',3''-trimethoxy-4',8''-oxy-8,8'-sesqui-neolignan-7''-O-β-D-glucopyranoside]* (**1**). Pale yellow oil. [α]^20^_D_: –29 (c = 0.79, MeOH); UV (MeOH) λ_max_ (logε) nm: 280 (3.32), 228 (3.68), 207 (4.03); CD (MeOH, nm) λ_max_ (Δε) 211 (+ 11.48), 229 (+ 1.30), 239 (− 3.74), 281 (+ 2.71), 297 (+ 1.37), 322 (+ 1.83); IR (KBr) ν_max_ 3364, 2935, 1603, 1513, 1453, 1423, 1369, 1268, 1224, 1155, 1127, 1075, 1029 cm^−1^; ^1^H- and ^13^C-NMR: see Table 1; HRESIMS *m/z*: 743.2863 ([M+Na]^ +^, C_36_H_48_O_15_Na^+^, calc. 743.2891).

### 3.4. Determination of the Absolute Configuration of Sugar Unit in **1**

A solution of **1** (1.0 mg), in 2 M aqueous CF_3_COOH (2.0 mL) was heated at 100 °C for 3 h in a water bath. The reaction mixture was diluted in H_2_O (4.0 mL) and extracted with EtOAc (4.0 mL × 3), then the aqueous layer was concentrated to remove CF_3_COOH. The residue was dissolved in pyridine (1.0 mL), to which L-cysteine methyl ester hydrochloride (2.0 mg) in pyridine (1.0 mL) was added. Then, the mixture was kept at 60 °C for 2 h. After the reaction mixture was dried *in vacuo*, the residue was trimethylsilylated with 1-trimethylsilylimidazole (0.2 mL) at 60 °C for 2 h in a water bath. Finally, the mixture was partitioned between hexane and H_2_O (0.3 mL each) and the hexane extract was analyzed by gas chromatography (GC) under the following conditions: column temperature, 250 °C; injection temperature, 250 °C; carrier N_2_ gas; flow rate 1.0 mL/min. In the acid hydrolysate of **1**, D-Glucose was confirmed by comparison of the retention times of their derivatives with those of D-glucose and L-glucose derivatives prepared in a similar way, which showed retention times of 13.66 and 14.34 min, respectively.

### 3.5. Enzymatic Hydrolysis of 1

A solution of **1 **(2.67 mg) in 0.1 M acetate buffer (pH 4.0, 2.0 mL) was treated with cellulase (3.0 mg) and then the reaction mixture was stirred at 40 °C for 12 h. The reaction mixture was diluted in H_2_O (4.0 mL) and extracted with EtOAc (4.0 mL × 3). After that, the EtOAc extract was further purified by semi-preparative HPLC (50% MeOH in H_2_O, flow rate 1.8 mL/min, wavelength 212 nm) to obtain compounds **1a** (1.2 mg, retention time 18.3 min).

*Erythro-(–)-secoisolariciresinol-sesquilignan* (**1a**)*.* Pale yellow oil. [α]^20^_D_: –7.1 (*c* = 0.028, MeOH); ^1^H-NMR (CD_3_OD, 400 MHz) *δ*_H_: 7.03 (1H, d, *J* = 1.8 Hz, 2''-H), 6.93 (1H, d, *J* = 8.2 Hz, 5''-H), 6.86 (1H, dd, *J* = 8.2, 1.8 Hz, 6''-H), 6.76 (1H, d, *J* = 8.1 Hz, 5-H), 6.68 (1H, d, *J* = 1.8 Hz, 2-H), 6.66 (1H, d, *J* = 8.0 Hz, 5'-H), 6.64 (1H, d, *J* = 1.9 Hz, 2'-H), 6.63 (1H, dd, *J* = 8.1, 1.8 Hz, 6-H), 6.54 (1H, dd, *J* = 8.0, 1.9 Hz, 6'-H), 4.88 (1H, d, *J =* 4.0 Hz, 7''-H), 4.20 (1H, m, 8''-H ), 3.86 (1H, d, *J =* 11.5 Hz, 9''-Ha), 3.82 (3H, s, 3''-OCH_3_), 3.77 (3H, s, 3-OCH_3_), 3.75 (3H, s, 3'-OCH_3_), 3.72 (1H, *J =* 11.5, 4.1 Hz, 9''-Hb), 3.69 (1H, overlapped, 9-Hb), 3.58 (2H, m, 9-Ha, 9'-Hb), 3.46 (1H, dd, *J* = 11.8, 5.2 Hz, 9'-Ha), 2.67 (2H, m, 7,7'-Ha), 2.60 (2 H, m, 7, 7'-Hb ), 2.06 (2H, m, 8,8'-H); HRESIMS *m/z*: 581.2344 ([M+Na]^ +^, C_30_H_38_O_10_Na ^+^, calc. 581.2363).

### 3.6. MTT Cytotoxicity Assay

Compound **1** was tested against five human cancer cell lines [HL-60 and SMMC-7721, A549 (lung), MCF-7 (breast), and SW480 (colon)] and a normal cell line (BEAS-2B). The antiproliferative assay was performed by the MTT colorimetric method as described previously [25]. Briefly, adherent cells were seeded into 96-well tissue culture plates with density of 1 × 10^5^ cells/mL. After 12 h, cells were treated with the medium containing different concentrations of test compounds for 48 h. Then, attached cells were incubated with MTT (15 μL, 5 mg/mL, 1 h) and subsequently solubilized in DMSO. The optical density of absorbency at 595 nm was then measured using a microplate reader. Experiments were performed in triplicate, and the values are the averages of three (n = 3) independent experiments. DDP (cisplatin, Sigma, San Francisco, CA, USA) and taxol were used as the positive control.

## 4. Conclusions

Studies carried out on the EtOH extracts of *U. sinensis*, revealed the presence of a new sesquilignan glucoside, urariasinoside A (**1**), was isolated from the aerial parts of *U. sinensis* together with eight known compounds, including two lignans (–)-syringaresinol (**2**), (–)-medioresinol (**3**); a sesquilignan (–)-(7*R*,7'*R*,7''*S*,8*S*,8'*S*,8''*S*)-4',4''-dihydroxy-3,3',3'',5,5'-pentamethoxy-7,9':7',9-diepoxy-4,8''-oxy-8,8'-sesquineolignan-7'',9''-diol (**4**); a dilignan (+)-(7*R*,7'*R*,7''*R*,7'''*R*,8*S*,8'*S*,8''*S*,8'''*S*)-4'',4'''-dihydroxy-3,3',3'',3''',5,5'-hexamethoxy-7,9':7',9-diepoxy-4,8'':4',8'''-bisoxy-8,8'-dineolignan-7'',7''',9'',9'''-tetraol (**5**), and four flavonoids derivatives 5,4'-dihydroxy-3'-methoxyflavanone-7-(6''-*O*-*β*-l-rhamnopyranosyl)-*β*-d-glucopyranoside (**6**), naringenin (**7**), quercetin (**8**), and kaempferol (**9**). Compounds **2**–**9** were isolated from *U. sinensis* for the first time. Cytotoxicity assays revealed that compound **1** was inactive (IC_50_ > 40 μM) against the HL-60, SMMC-7721, A549, MCF-7, and SW480 cell lines.

## Supplementary Materials

Supplementary materials can be accessed at: http://www.mdpi.com/1420-3049/19/1/1178/s1.
