# Supplementary material for: A New Sesquilignan Glucoside from Uraria sinensis
Source: Molecules. 2014 Jan 17;19(1):1178–88. doi: 10.3390/molecules19011178 (PMC6271682; doi:10.3390/molecules19011178)

Supporting Information

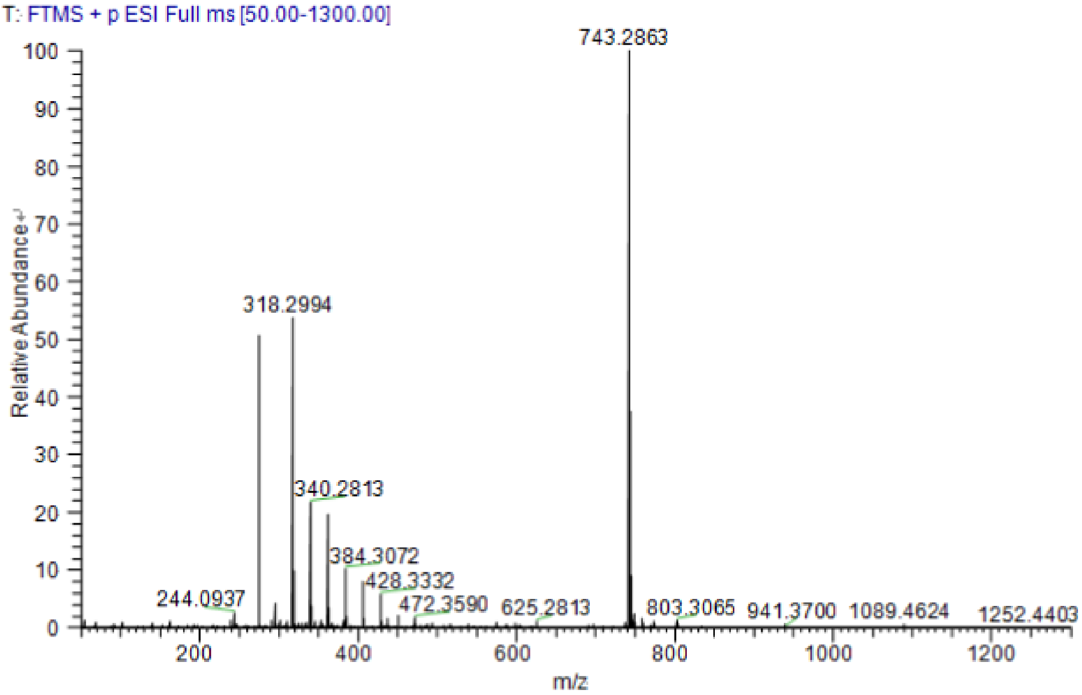

Figure S1. HRESIMS spectrum of compound **1**.

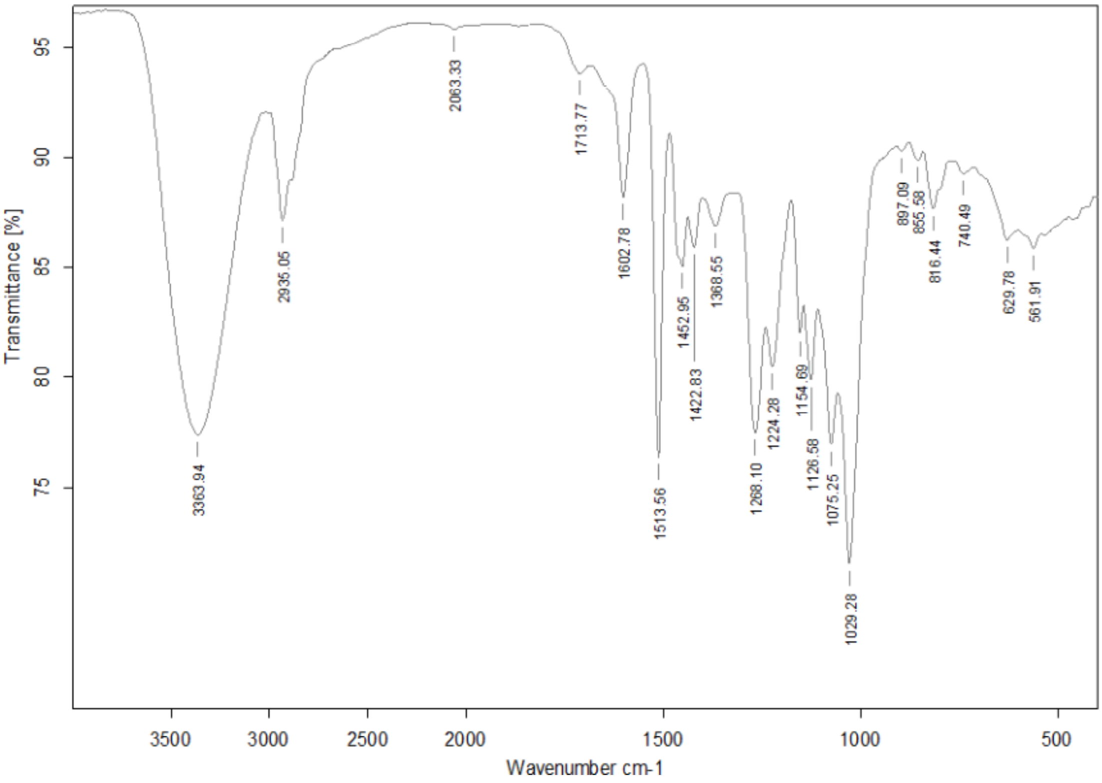

Figure S2. IR spectrum of compound **1**.

Figure S3. UV spectrum of compound **1**.

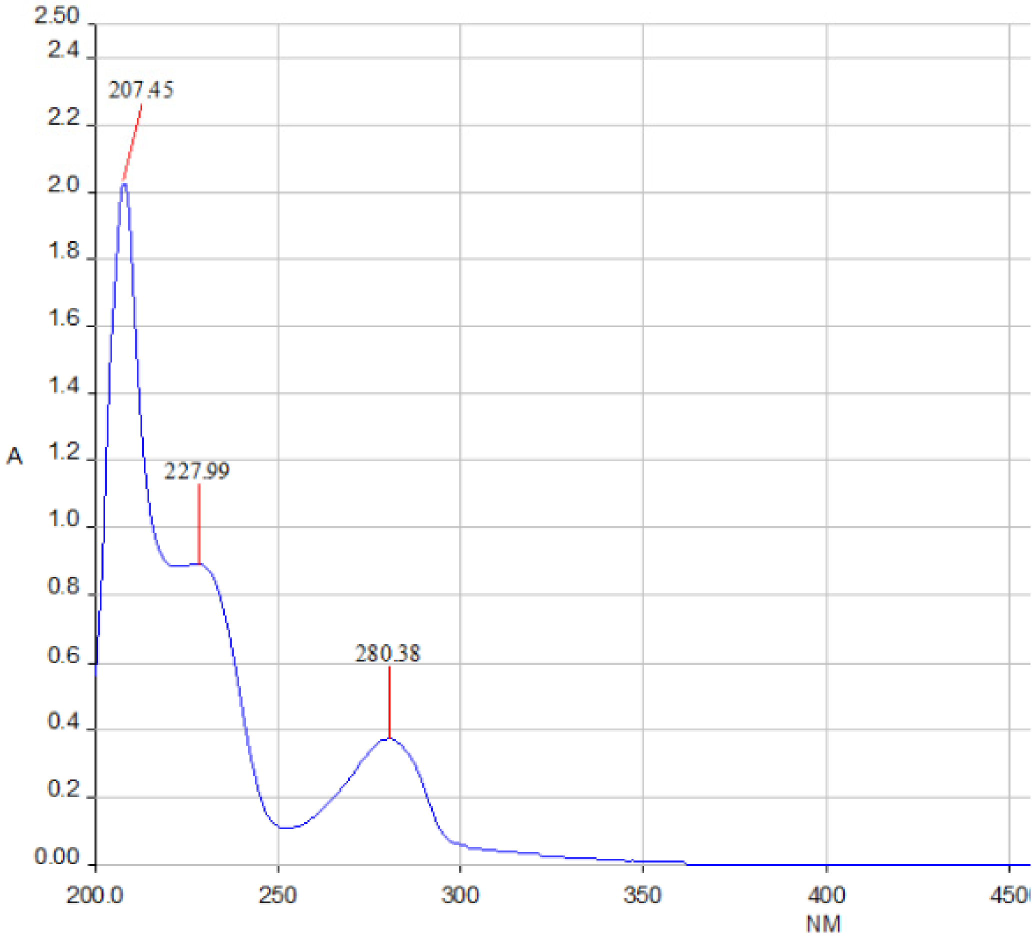

Figure S4. ^{1}H-NMR spectrum of **1**.

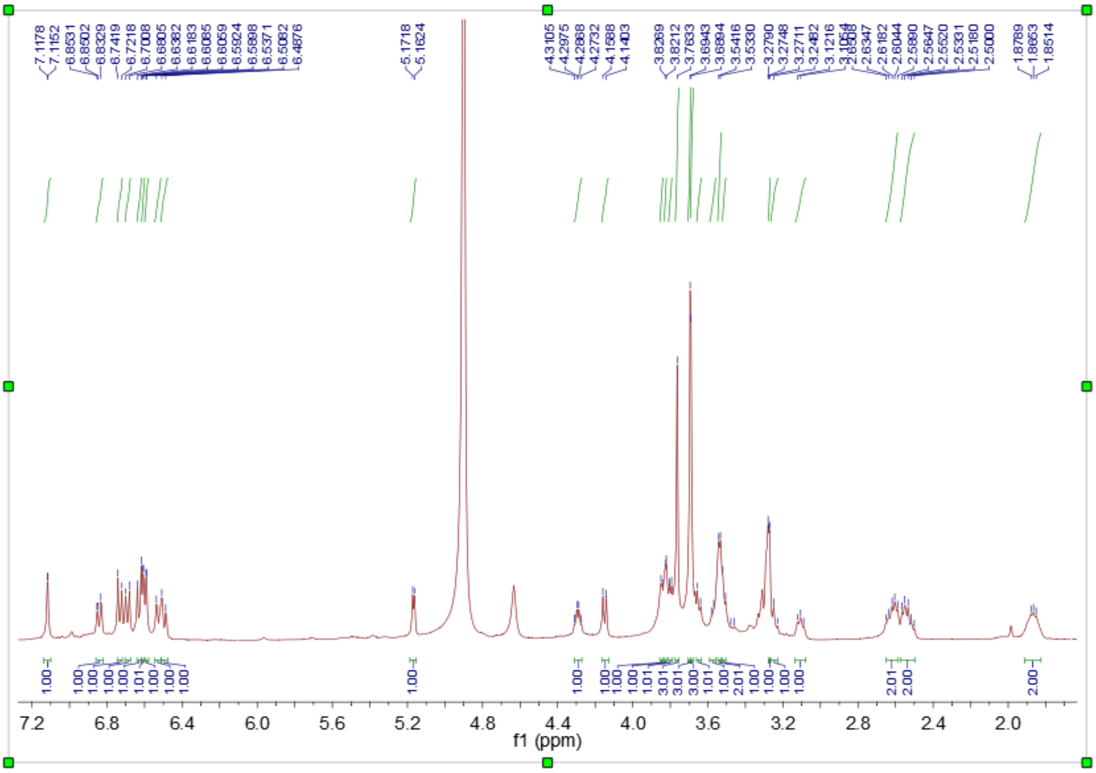

Figure S5. ^{13}C-NMR and DEPT spectra of compound **1**.

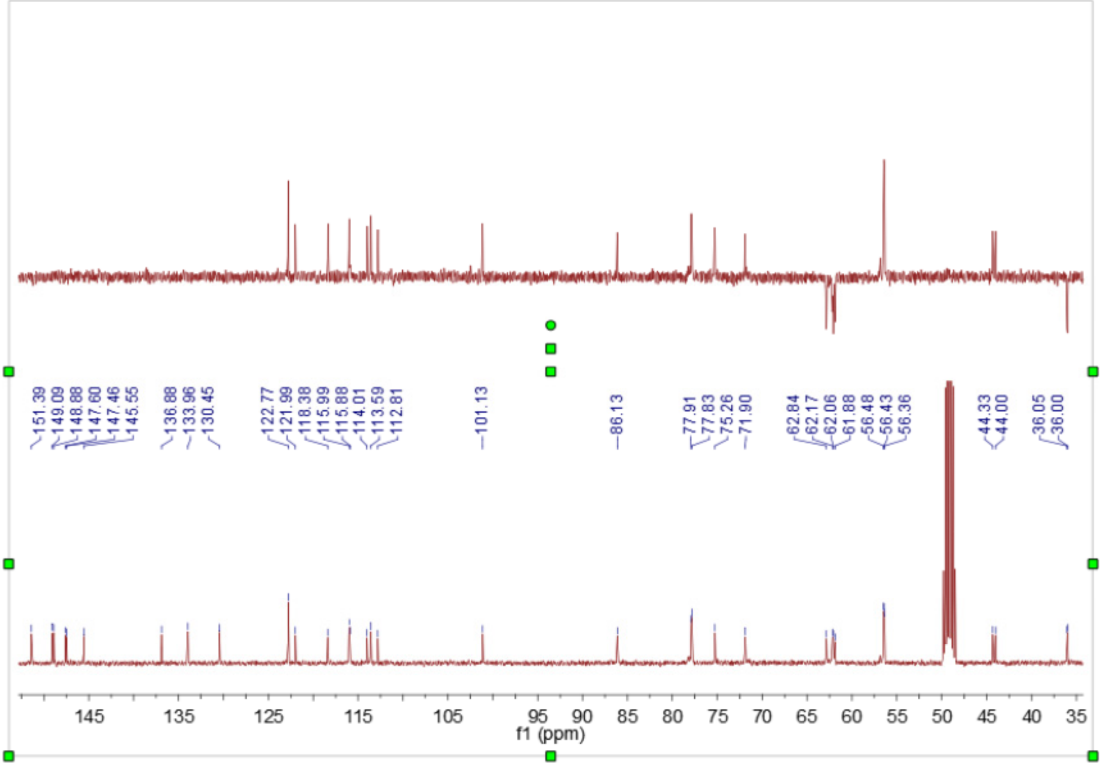

Figure S6. HSQC spectrum of compound **1**.

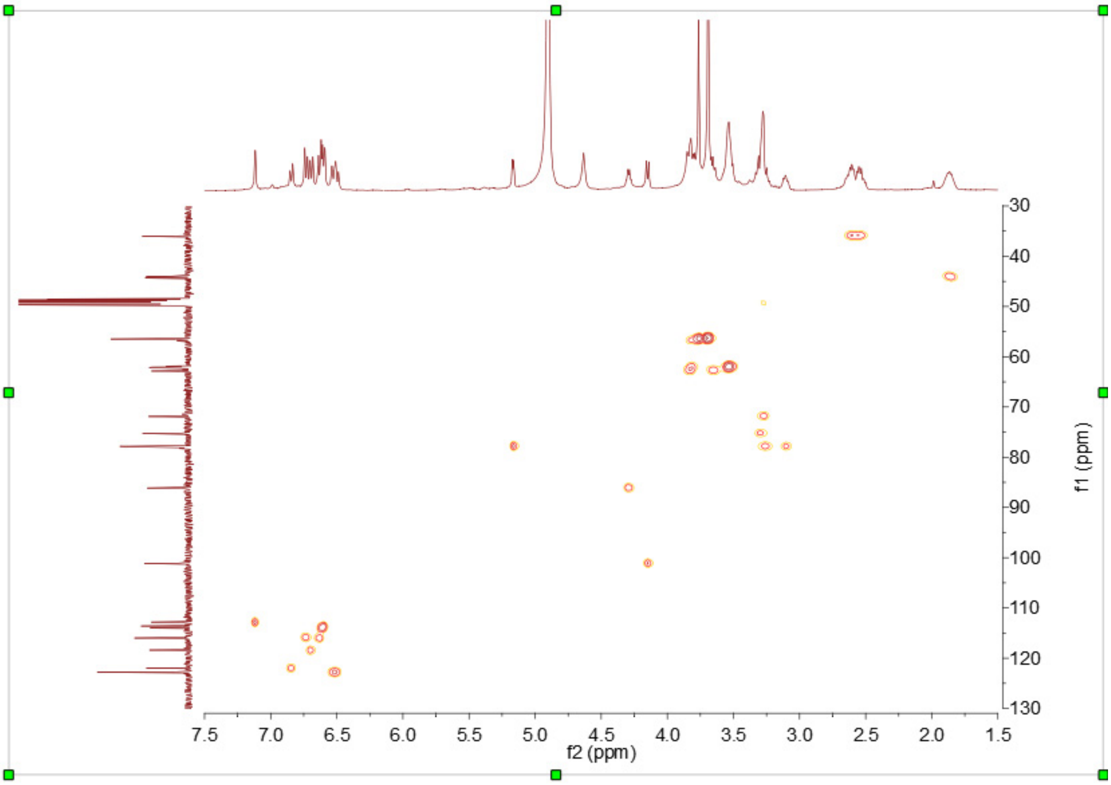

Figure S7. COSY spectrum of compound **1**.

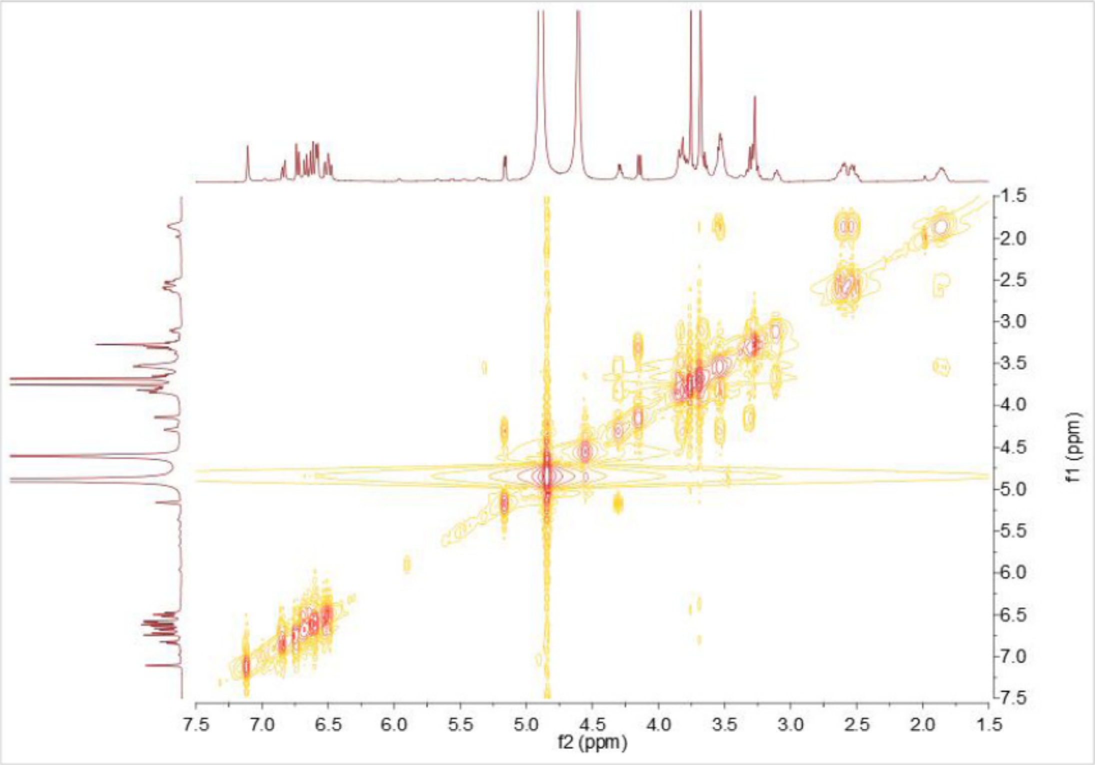

Figure S8. HMBC spectrum of compound **1**.

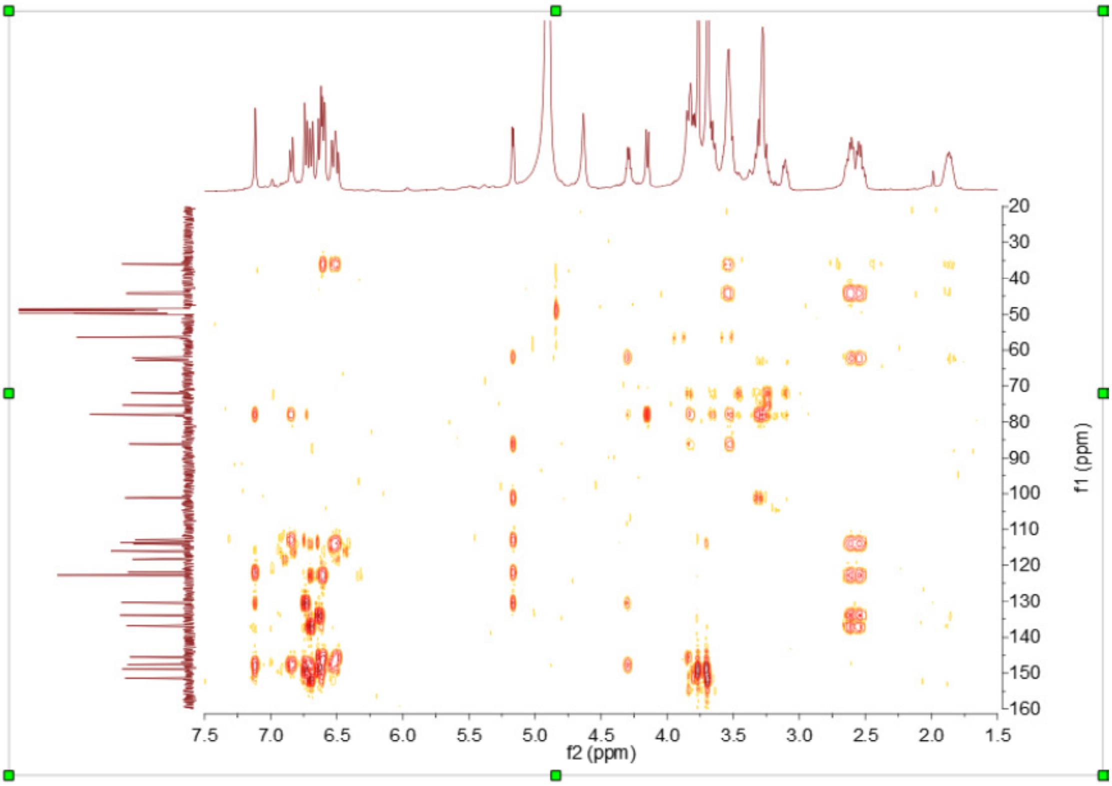

Figure S9. NOSEY spectrum of compound **1**.

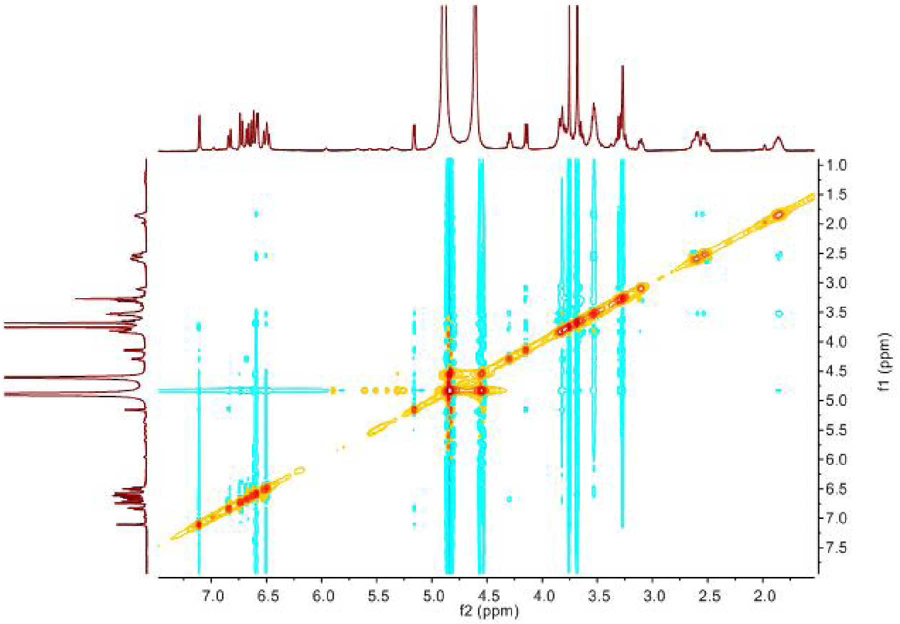

Figure S10. GS analysis of the sugar of compound **1**. (**A**) L-glucose; (**B**) D-Glucose; and (**C**) sugar of compound **1**.

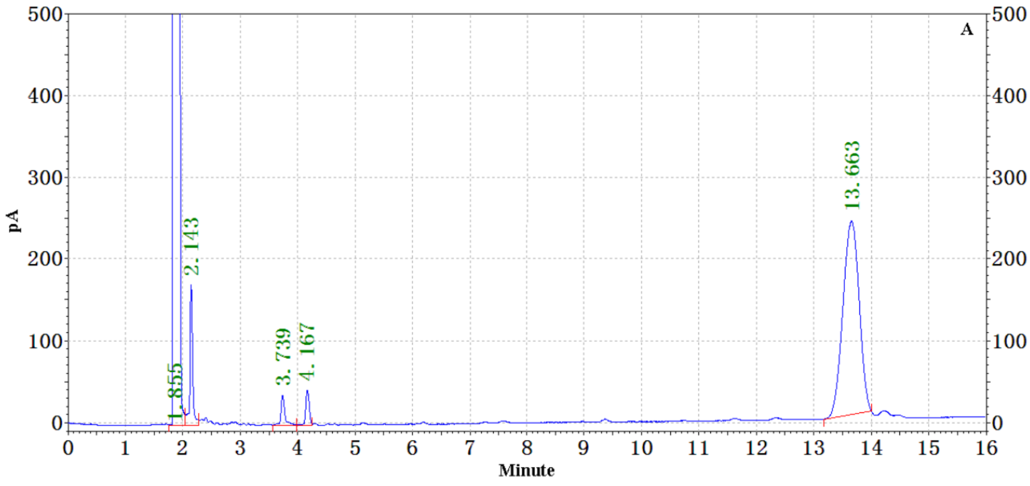

(**A**)

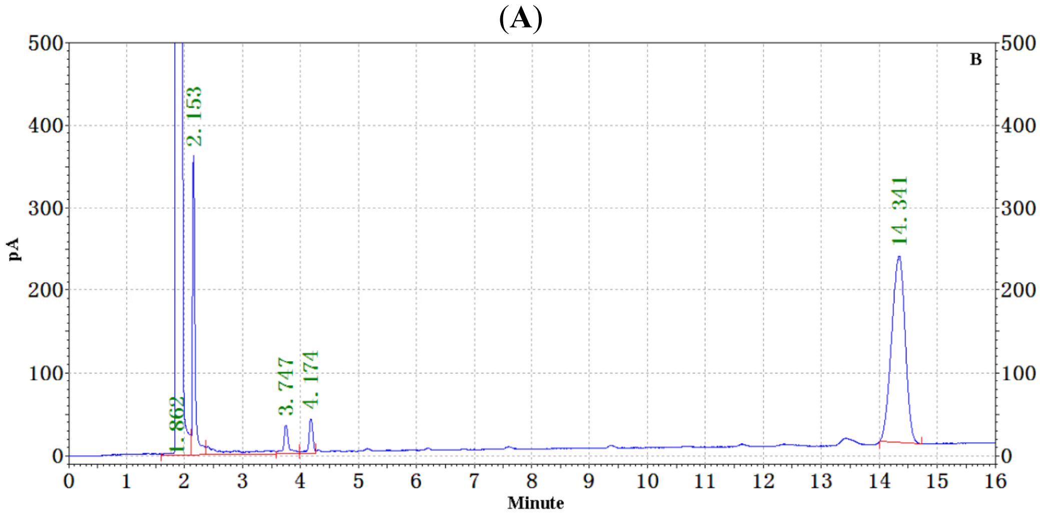

(**B**)

Figure S10. *Cont.*

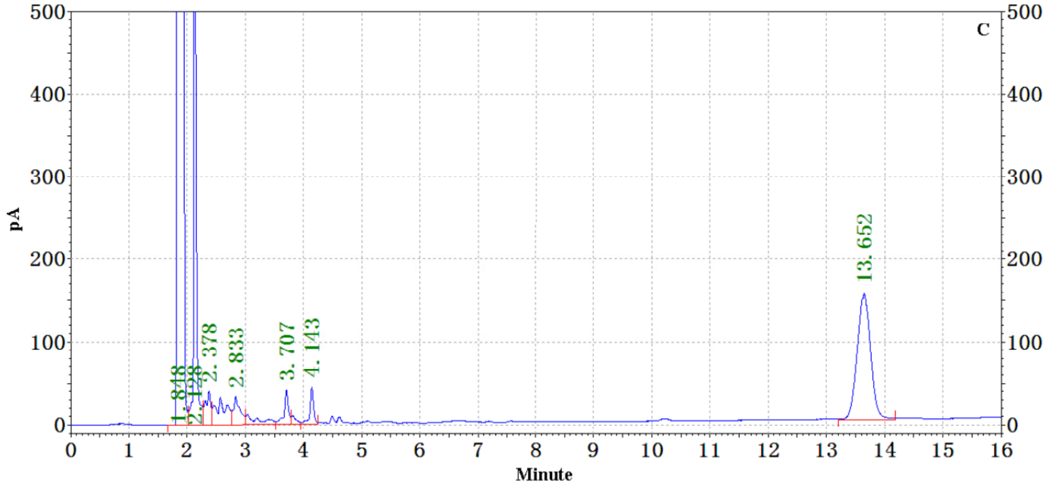

(**C**)

Figure S11. The CD Spectrum of 1 in MeOH.

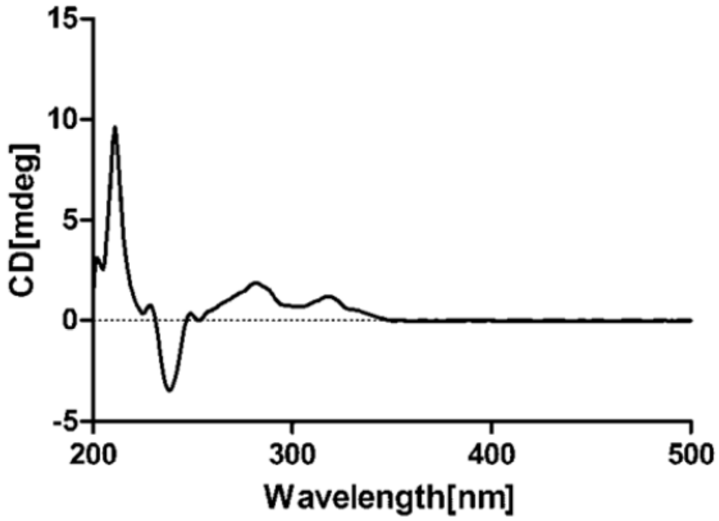

Figure S12. The HRESIMS spectrum of compound **1a**.

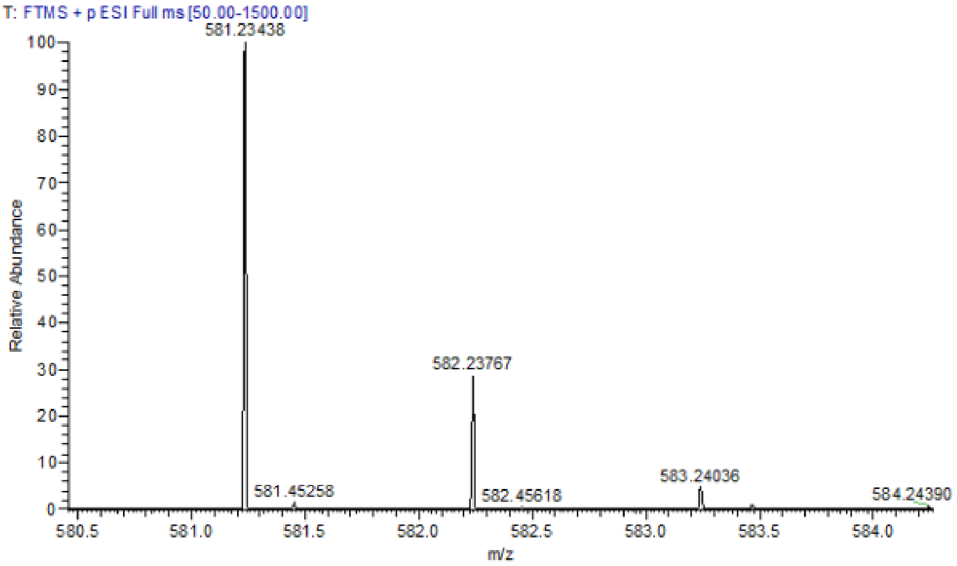

Figure S13. The ^{1}H-NMR spectrum of compound **1a**.

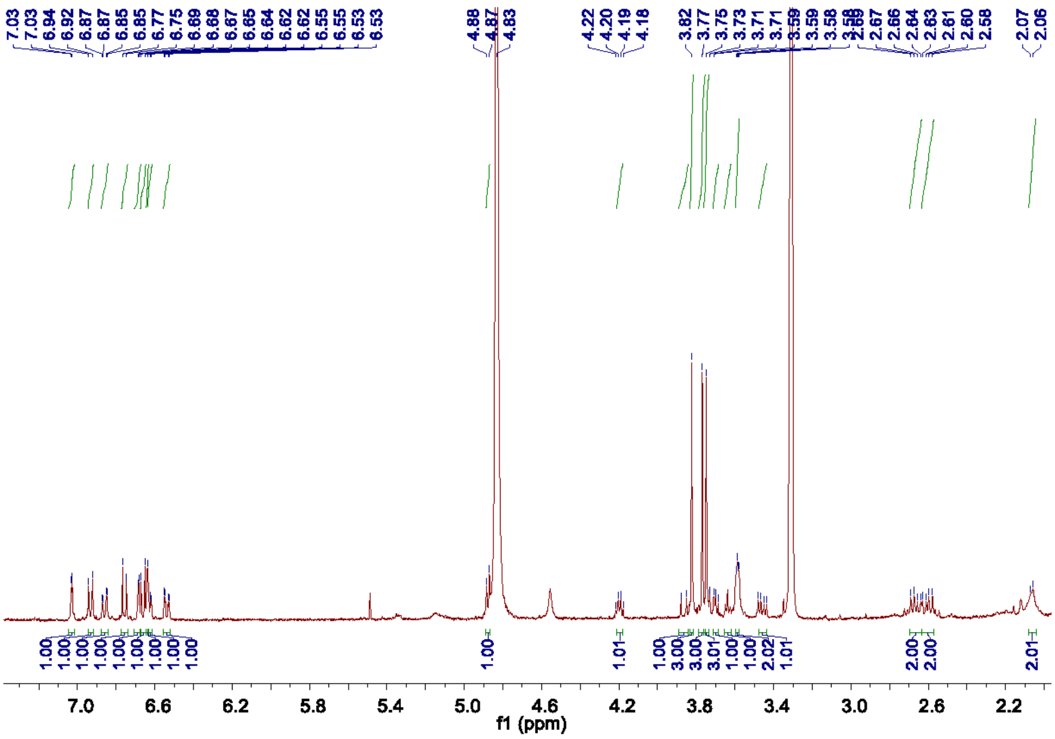

Figure S14. The expanded ^{1}H-NMR spectrum of **1**.

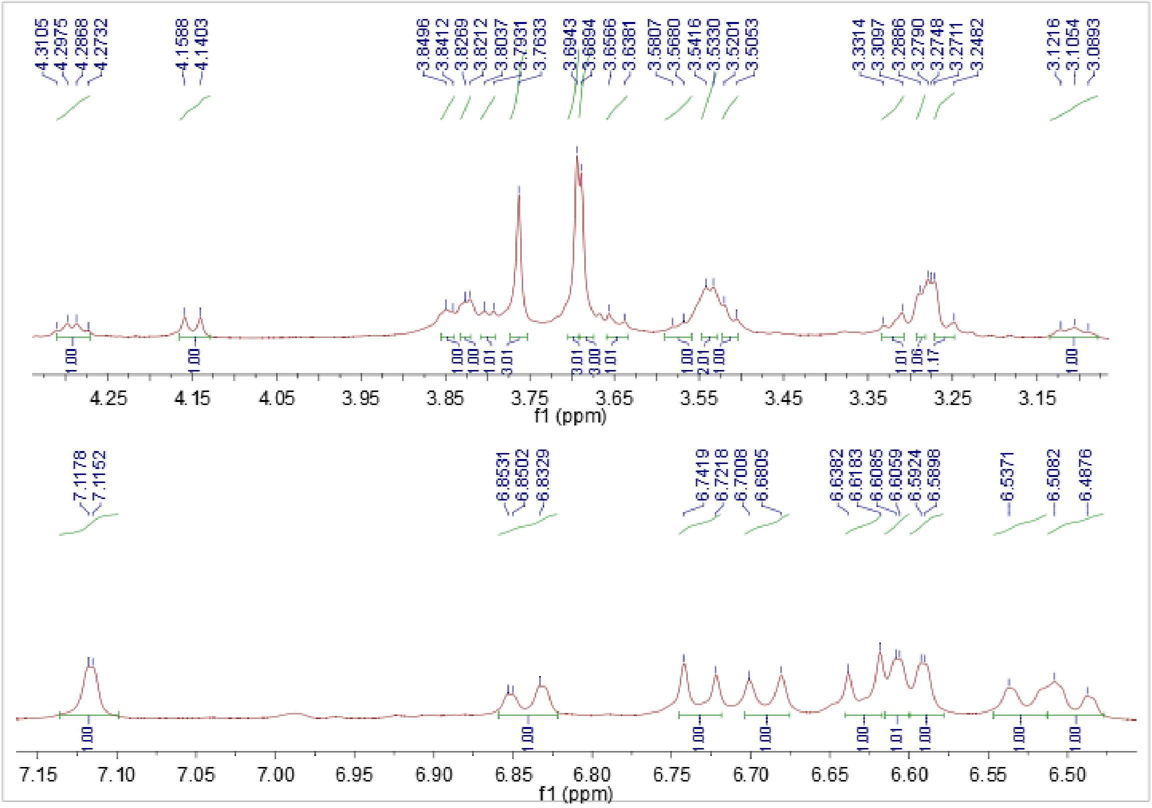

Supplement: Supplementary file 1 [file molecules-19-01178-s001.pdf]
